# Epidemiology of nummular eczema – methodological approaches and outcomes from nationwide claims data analyses

**DOI:** 10.1111/ddg.15932

**Published:** 2025-11-16

**Authors:** Kristina Hagenström, Katharina Müller, Theresa Klinger, Charlotte Willers, Kilian Eyerich, Matthias Augustin

**Affiliations:** ^1^ German Center for Health Services Research in Dermatology (CVderm) Institute for Health Services Research in Dermatology and Nursing (IVDP) University Medical Center Hamburg‐Eppendorf (UKE) Hamburg Germany; ^2^ Dept. of Dermatology and Venereology University Medical Center of Freiburg Freiburg Germany

**Keywords:** Atopic dermatitis, frequency of illness, incidence, prevalence, severity, statutory health insurance data, validation

## Abstract

**Background and Objectives:**

Nummular eczema (NE) is a chronic skin condition with limited epidemiologic data. This study estimates its prevalence, incidence and severity in Germany.

**Methods:**

A cross‐sectional analysis (2016–2022) used German insurance claims data to identify NE cases (ICD‐10 L30.0) and assess severity (hospitalization, work disability, systemic treatment) and comorbid skin conditions, including atopic dermatitis (AD).

**Results:**

In 2022, the prevalence of NE ranged from 0.07% to 0.26%. The incidence was 0.16%–0.17%. The mean age was 47.5 years, and 57.4% of the patients were male. Older males had a higher prevalence, while children younger than six years (0.34%), especially those younger than 2 years (0.56%–0.57%), were more affected. Severe NE was found in 14.8% of the patients. AD co‐occurred in 18% of NE cases. NE patients had an increased risk of lichen simplex chronicus (RR 9.71), irritant contact dermatitis (RR 9.60), pruritus (RR 5.71), allergic rhinitis (RR 1.78), and allergic asthma (RR 1.49).

**Conclusions:**

Despite differences in methodological approaches leading to some variation, the prevalence of NE in the German population can be reasonably estimated at approximately 0.26%. Overlaps and miscoding may have occurred, particularly with other forms of eczema, underscoring the need for reliable diagnostics and standardized coding.

## INTRODUCTION

Nummular eczema (NE) is a mostly chronic, recurrent and pruritic eczematous skin disease occurring in both children and adults. It is distinct from other types of eczema, based on phenotypical appearance in correlation with histological findings. A codominant Th2/Th17 immune response pattern is observed, that suggest NE and atopic dermatitis (AD) share a common inflammatory pathway.^1^ Clinically, it is characterized by inflammatory round or oval erythematous and eczematous plaques on usually dry skin.^1^, ^2^ Typically, the lesions are multiple, and the size varies from about 1 cm to 6 cm in diameter. Although being frequent in dermatology routine care, it is an under‐researched condition with only few publications on the epidemiology.

It has been demonstrated that NE is observed in 13.5% of individuals with atopic dermatitis,^3^ in 3.5% of all dermatological diseases or eczematous dermatitis^4^, ^5^ or in 7% of persons with hand eczema.^6^ To date, no epidemiological data have been published in Germany. Furthermore, there has been a low volume of published work on the prevalence and incidence of NE worldwide. It has been demonstrated that NE prevalence increases with age.^5^, ^7^, ^8^ Men are predominantly affected between the ages of 50 and 65, while women in particular are affected between the ages of 15 and 25. The diagnosis is mainly raised clinically based on the typical discoid‐shaped lesions. The histopathological appearance is not specific for this type of eczema. Thus, other clinically resembling skin diseases should be excluded.^9^ Overlap with other dermatological diseases, including contact dermatitis and atopic dermatitis has been reported.^7^, ^10^

In the light of limited literature, new population‐based data are of great importance for understanding the disease burden of NE, particularly for appropriate health care planning. The present study fills this scientific gap by providing a comprehensive and longitudinal insight into NE epidemiology in Germany, based on a large, unselected population from a nationwide statutory health insurance. A second aim of this claims data study was to develop a concept for the internal validation of the data as a basis for valid estimation of NE prevalence, incidence and severity in Germany.

## METHODS

### Study design and data source

Statutory health insurance (SHI) is an essential part of the German health care system: about 89% of the German population (about 72 million persons) is insured by one of the 95 statutory health insurance funds. The remaining 11% are privately insured (Health reporting of the Confederation [GBE‐Bund]). The DAK‐Gesundheit (DAK‐G) is a large and nationwide operating health insurance company, with 5.5 million members respectively in 2022. The study population of the DAK‐G analyzed in this project is an anonymized 40% random samples of all insured persons who were insured for at least one day between *(1)* 2016 and 2020 and *(2)* 2018 and 2022 (54.9% women, average age 46.5 years in 2022). The legal basis and data protection of the sample were subject to the German Social Code (SGB) and the Federal Data Protection Act (BDSG). The data from DAK‐G have been shown to be representative for the total German population after adjusting for age and gender.^11^

The SHI data contain all billing‐relevant information from the outpatient and inpatient sectors, including work incapacity and all prescription drugs in the outpatient sector, as well as all outpatient contacts with doctors, coded diagnoses, billed services and the time specification of the physician's visit at the quarterly level.^12^ Overall, the SHI data reflect the coding prevalence of a given disease, and persons untreated within the SHI system are not detected.

### Case definition and covariates

For internal validation, the prevalence and incidence of NE were determined using different case definitions. Identification of individuals with NE was based on the ICD‐10‐GM code L30.0 during either outpatient care or inpatient admission. For the incidence, different diagnosis‐free periods (wash‐out periods) were defined (Table 1). The study population had to be continuously insured during the observed years (at least 1 day per quarter). Persons who died during the observation period were not excluded from the analyses.

**TABLE 1:** Case definitions to determine the prevalence and incidence of nummular eczema (NE; ICD‐10‐GM L30.0) in German claims data.

	Case definition (prevalence)			Case definition (incidence)		
Criteria	A (≥ 1 NE dx) Base case definition	B (≥ 2 NE dx within 1 year)	C (≥ 2 NE dx within 3 years)	I (2 years dx‐free)	II (3 years dx‐free)	III (4 years dx‐free)
≥ 1 inpatient principal or secondary NE dx	**+**	**+**	**+**	**+**	**+**	**+**
OR						
≥ 1 confirmed outpatient dx	**+**			**+**	**+**	**+**
≥ 1 confirmed outpatient dx in at least 2 quarter		**+**				
≥ 1 confirmed outpatient dx in at least 1 quarter of the observation year and at least 1 out of the of the previous 2 years)			**+**			
AND						
dx‐free period (outpatient and inpatient) for prior 8 quarters				**+**		
dx‐free period (outpatient and inpatient) for prior 12 quarters					**+**	
dx‐free period (outpatient and inpatient) for prior 16 quarters						**+**

*Abbr*.: +, included in case definition; dx, diagnosis

In addition, the concomitant occurrence of AD (ICD‐10‐GM L20) with NE was investigated. A sensitivity test was conducted to determine the epidemiological range of individuals with NE who were diagnosed with AD. For this purpose, individuals with AD were included who had no NE in the prevalence year but had a diagnosis of NE within the 2 years before or after the prevalence year. The following ICD‐10‐GM codes were analyzed to estimate other concomitant skin and atopic diseases in people with NE and compared with people without NE (online supplementary Table S1).

Since claims data do not provide sufficient clinical information to characterize the severity disease, surrogate markers are used. Severity of NE was thus operationalized through at least one of the following: *(1)* the need of hospital stay (inpatient principal NE diagnosis), *(2)* periods of days off work, *(3)* use of systemic drugs (online supplementary Table S2). Systemic antibiotics and antihistamines were excluded from the set of surrogate markers as they are frequently employed for the treatment of a range of illnesses, rendering them less disease‐specific.

### Statistical analysis

The administrative estimates of annual prevalence and incidence rates are reported as percentages with their corresponding 95% confidence intervals (CI) for the observation years 2016 to 2022. All results were standardized by age and sex for the German population as of 31 December of the respective year according to the German federal statistics office *Destatis* (direct standardization). Rates were stratified by age, sex and regional distribution (federal state). Age was stratified into ten age groups and further subgroups (development phase in years: 0 ≤ 6, 6 ≤ 12, 12 ≤ 18, 18 ≤ 25, 25 ≤ 50, > 50 and age up to 18 years). Descriptive statistical methods were used to describe the data. Differences between the concomitant diseases of the respective populations considered (NE vs. without NE) were presented using rate ratios (RR) with the respective 95% CI. All analyses were performed using the SAS for Windows^®^ software package, version 9.5 (SAS Institute Inc., Cary, North Carolina, USA).

## RESULTS

In 2022, 6,431 of the 2,366,437 insured individuals exhibited NE, which corresponds to a standardized rate of 0.26% (base case definition A, ≥ 1 NE diagnosis) (Table 2). Extrapolating to the German population, there would be an estimated 220,988 individuals diagnosed with NE. The mean age for people with NE was 47.5 (standard deviation [SD] 25.3, median 50) and 57.4% were men. In more conservative case definitions, the prevalence rate was found to be considerably lower at 0.07% (case definition B, ≥ 2 NE diagnosis within one year) and 0.11% (case definition C, ≥ 2 NE diagnosis within three years). In the most conservative scenario, the total number of persons with NE in Germany would be about 56,000. The prevalence rates exhibited a slight decline from 2016 to 2022, with the exception of case definition B and C (≥ 2 NE diagnosis).

**TABLE 2:** Standardized prevalence of nummular eczema (NE) according to different case definitions within the observational years.

Year	N	Case definition A (base case) (≥ 1 NE dx)				Case definition B (≥ 2 NE dx within 1 year)				Case definition C (≥ 2 NE dx within 3 years)			
		*n*	*Rate,%*	*95% CI*	*Extrapolated to German population*	*n*	*Rate,%*	*95% CI*	*Extrapolated to German population*	*n*	*Rate,%*	*95% CI*	*Extrapolated to German population*
2016	2,331,615	7,643	0.30	0.30–0.30	250,400	1,959	0.07	0.07–0.07	61,186	–	–		–
2017	2,306,774	7,487	0.30	0.30–0.31	252,285	1,896	0.07	0.07–0.07	61,589	–	–		–
2018	2,276,281	7,189	0.29	0.29–0.30	244,655	1,895	0.08	0.07–0.08	62,566	1,949	0.08	0.08–0.08	64,247
2019	2,246,743	7,089	0.30	0.29–0.30	246,175	1,865	0.07	0.07–0.08	62,082	2,822	0.11	0.11–0.11	93,741
2020	2,232,255	6,544	0.27	0.27–0.27	226,987	1,727	0.07	0.07–0.07	58,811	2,858	0.12	0.12–0.12	96,587
2021	2,336,344	6,829	0.28	0.28–0.28	234,990	1,805	0.07	0.07–0.07	61,042	2,938	0.12	0.12–0.12	100,075
2022	2,366,437	6,431	0.26	0.26–0.26	220,988	1,689	0.07	0.07–0.07	56,984	2,779	0.11	0.11–0.11	93,283

*Abbr*.: dx, diagnosis; CI confidence interval

The data indicate that incidence is only mildly affected by an extended washout period, decreasing from 0.17% with case definition I (2‐year diagnosis‐free) to 0.16% with case definitions II and III (3‐ and 4‐year diagnosis‐free) (Table 3). Furthermore, a slight decrease in incidence was observed according to case definitions I and II (2 years and 3 years diagnosis‐free), while the incidence according to case definition III (4 years diagnosis‐free) was 0.16% in the observed years.

**TABLE 3:** Standardized incidence of nummular eczema (NE) according to the different incident case definitions, without and with a concomitant atopic dermatitis (AD) within the observational years.

Year	Incident case definition I (base case) (2 years dx‐free)					Incident case definition II (3 years dx‐free)					Incident case definition III (4 years dx‐free)				
	N	n	Rate,%	95% CI	Extrapolated to German population	N	n	Rate,%	95% CI	Extrapolated to German population	N	n	Rate,%	95% CI	Extrapolated to German population
2018	2,135,290	4,571	0.20	0.20–0.20	163,373	‐	‐	‐		‐	‐	‐	‐		‐
2019	2,120,101	4,574	0.20	0.20–0.20	166,680	2,052,159	4,253	0.19	0.19–0.19	157,969	‐	‐	‐		‐
2020	2,108,558	4,113	0.18	0.18–0.18	149,102	2,048,737	3,816	0.17	0.17–0.17	140,073	1,983,698	3,614	0.16	0.16–0.16	135,569
2021	2,107,591	4,124	0.18	0.18–0.18	152,877	2,040,477	3,835	0.17	0.17–0.18	145,233	‐	‐	‐		‐
2022	2,138,829	3,881	0.17	0.17–0.17	144,138	2,034,954	3,573	0.16	0.16–0.16	137,182	1,971,391	3,356	0.16	0.15–0.16	131,189

*Abbr*.: dx, diagnosis; CI confidence interval

**FIGURE 1:**
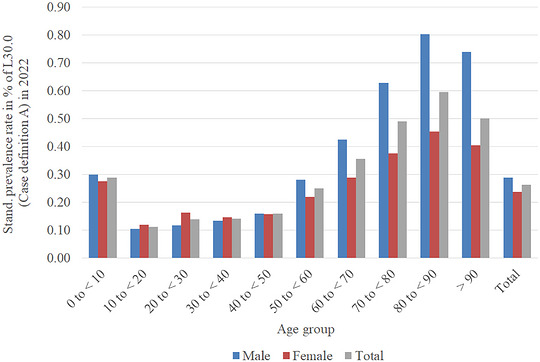
Standardized prevalence (base case definition A [≥ 1 diagnosis of NE]) of NE by age and sex in 2022 in percent.

**FIGURE 2:**
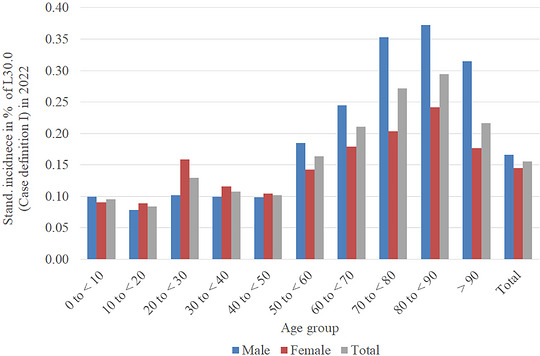
Incidence rates (incident base case definition I [diagnosis‐free period of 16 quarters, outpatient and inpatient]) of NE by age and sex in 2022 in percent.

The prevalence and incidence of NE increases with age, with a higher prevalence among men, particularly in the older age group (Figures 1 and 2). Higher rates were observed in the older age group, where males with NE demonstrated a pronounced upward trajectory, reaching a standardized prevalence rate of 0.80% and incidence rate of 0.37%. In contrast, the prevalence rate for females with NE was 0.45%. The age and sex distributions were found to be consistent across the various case definitions, with no discernible changes observed over time.

**FIGURE 3:**
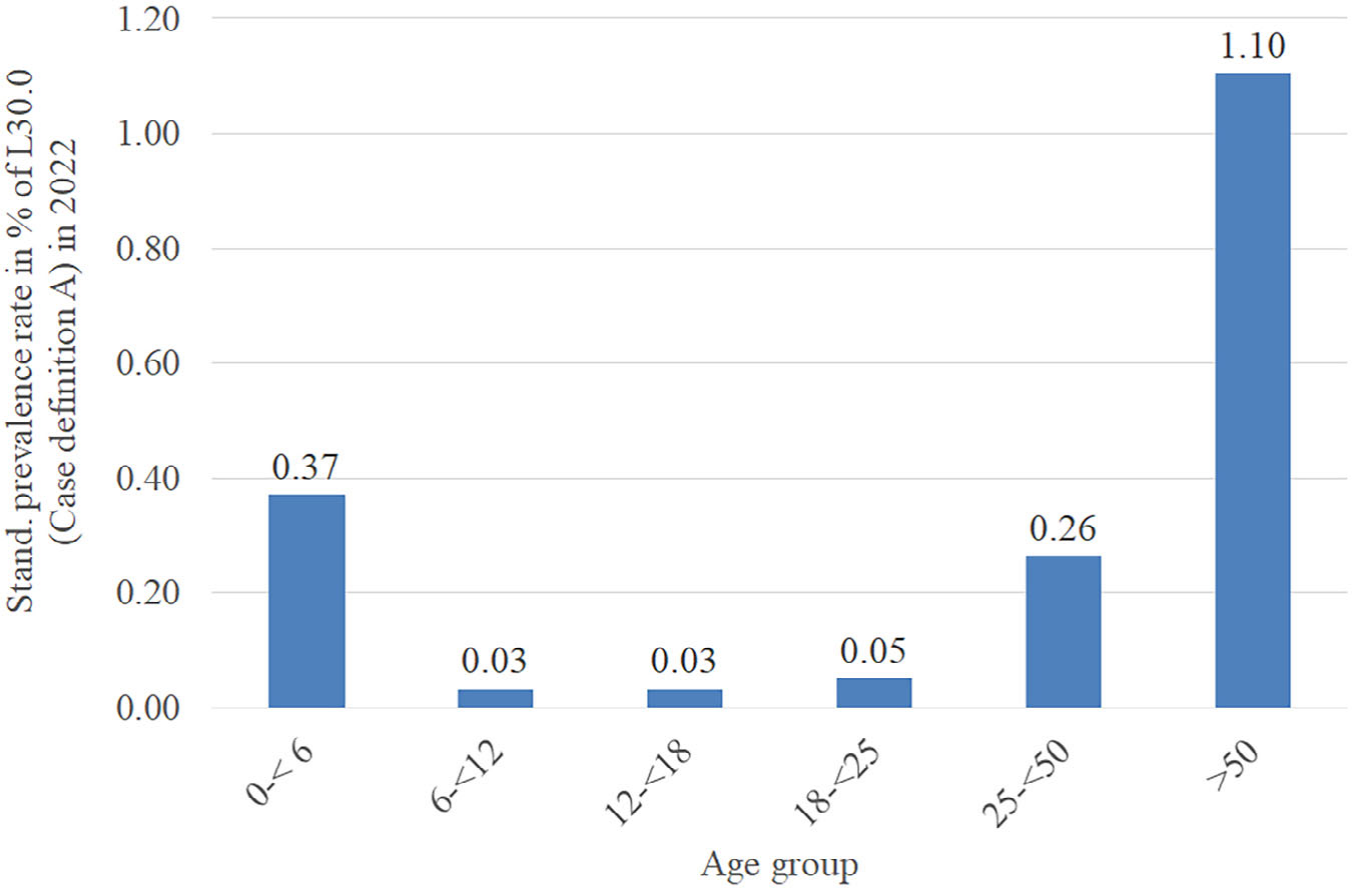
Standardized prevalence of nummular eczema (NE) in persons up to 18 years in 2022 (base case definition A [≥1 diagnosis of NE]) in percent.

**FIGURE 4:**
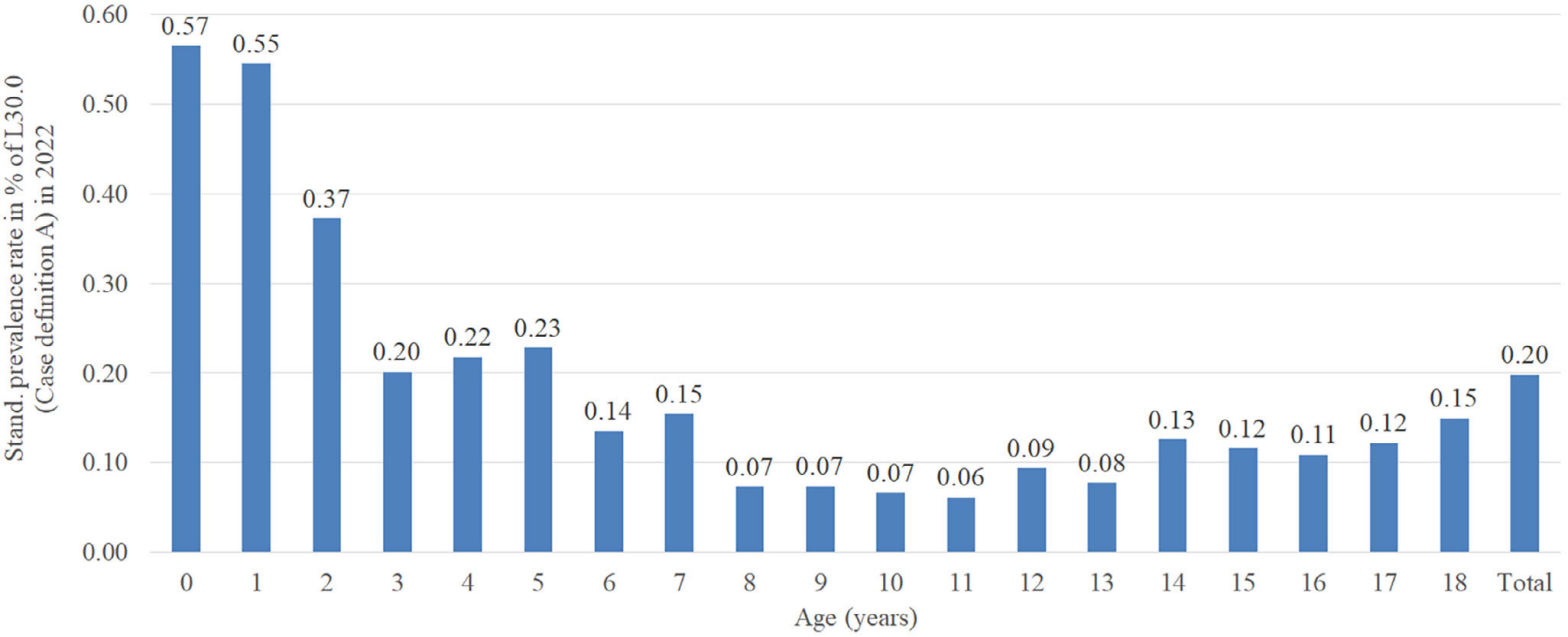
Standardized prevalence of nummular eczema (NE) (base case definition A [≥1 diagnosis of NE]) by age group (development phase) in 2022 in percent.

A further age classification according to developmental phase demonstrates that children under the age of 6 (Figure 2, 4) and particularly under 2 years (Figure 3) are particularly affected of NE.

**FIGURE 5:**
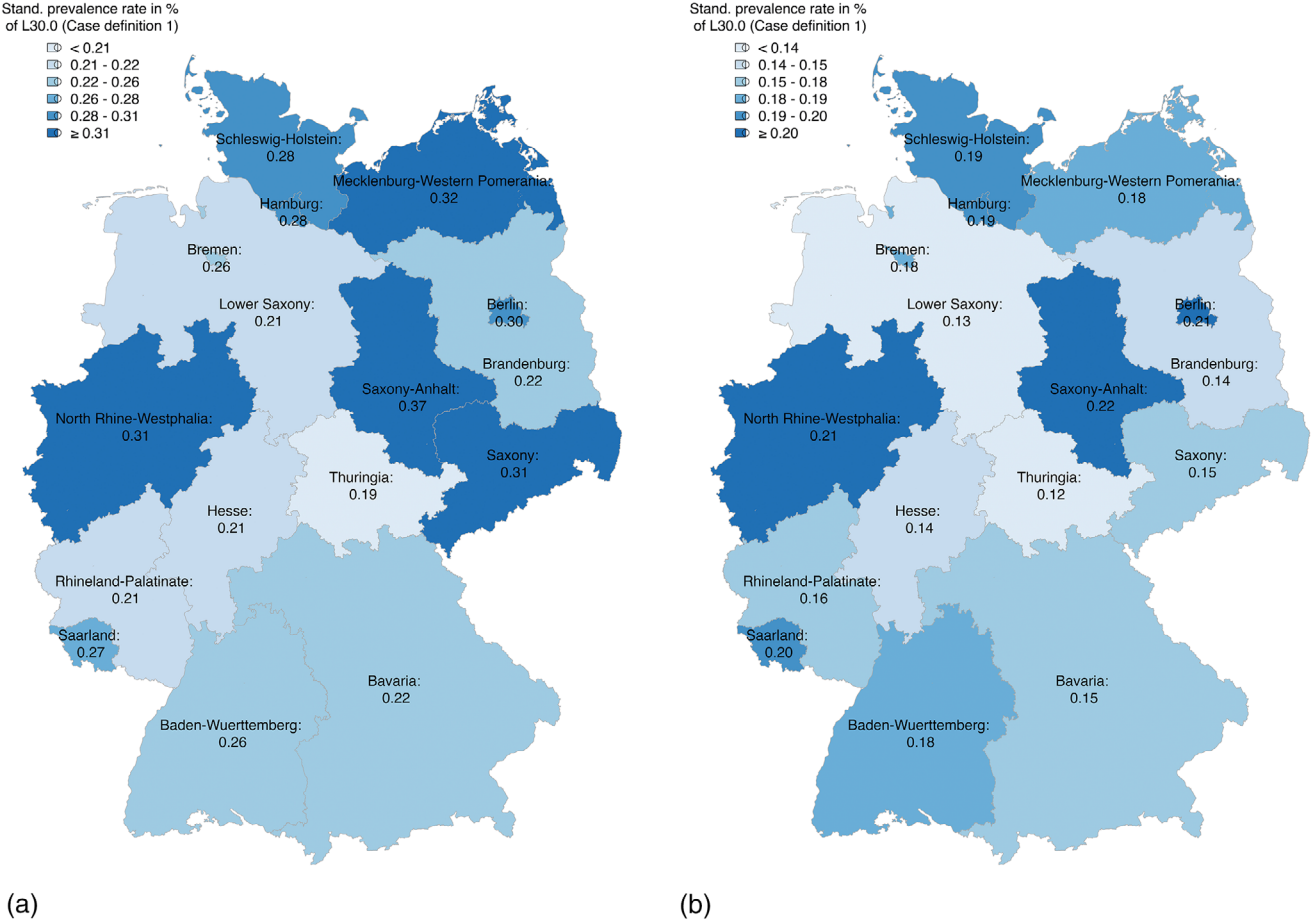
Standardized prevalence (a) base case definition A (≥ 1 diagnosis of NE) and (b) incidence: base case definition I (4 years diagnosis‐free) of nummular eczema (NE) in 2022 by federal state in Germany.

Marked regional variations in the prevalence and incidence of NE were observed across Germany. In 2022, the highest prevalence and incidence rates were observed in the federal states of Saxony‐Anhalt and North Rhine‐Westphalia. In contrast, the lowest rates were recorded in Thuringia (Figure 5).

Moreover, 18.0% of individuals with an NE also received an AD diagnosis in the same year (in accordance with base case definition A, ≥ 1 NE diagnosis). A comparison of the age and sex distribution of individuals diagnosed with NE and AD or without AD reveals that AD co‐diagnosed was identified especially in childhood (0–10) (online supplementary Figure S1). Sensitivity analysis showed that among individuals with AD (prevalence 4.05%) (supplementary Figure S2) who had no NE in the prevalence year, 1.76% were diagnosed with NE within the 2 years before or after the prevalence year. The prevalence of concurrently coded skin and atopic diseases demonstrated that people with NE had a higher risk of developing these conditions compared to people without NE. For example, individuals with NE had a higher risk of developing exfoliative dermatitis (RR 11.0, CI 3.6–33.5), lichen simplex chronicus (RR 9.7, CI 8.5–11.2), irritant contact dermatitis (RR 9.6, CI 7.4–12.4), unspecified contact dermatitis (RR 7.0, CI 5.9–8.3), seborrheic dermatitis (RR 6.0, CI 5.4–6.6), and pruritus (RR 5.7, CI 5.2–6.2) compared with those without NE (online supplementary Table S2).

In 2022, 14.8% of insured persons with NE were classified as having severe disease according to case definition A (≥ 1 NE diagnosis) (online supplementary Table S3). The percentage was slightly higher, at 15.3% according to case definition C (≥ 2 NE diagnosis within 3 years). A high proportion of people diagnosed with NE were receiving systemic medication, ranging from 92.1% to 97.4%. The hospitalization rate was less than 10%, while the proportion of people off work due to NE was less than 3%.

## DISCUSSION

The objective of this claims data analysis was to provide initial robust data on the epidemiology of NE in the German population. The diagnosis code used (ICD‐10‐GM L30.0) is specific to nummular eczema. However, beyond ICD‐10 coding, further verification of NE was not possible, for example by medication, since no specific treatment for NE currently exists that could improve diagnostic specificity.^13^ Different case definitions were investigated in order to identify the sensitivity of the primary case criteria in these health insurance data. Furthermore, the presence of the diagnosis code in different time periods was assessed to also test for the robustness of data. The prevalence of the condition was observed to range between 0.11% and 0.30% in 2019 (depending on the case definitions). Prevalence by base case definition A (≥ 1 NE diagnosis) was slightly lower during the period of the COVID pandemic period between 2020 and 2023. The prevalence of NE is underestimated by case definitions that include patient visits or prescriptions in more than one quarter, as the disease often manifests in relapses,^9^, ^14^ which in some patients necessitates only episodic treatment. Therefore, the criterion of at least two diagnoses in two different quarters of a year (case definition B, ≥ 2 NE diagnosis) is too strict, and two diagnoses within a longer period of three years (case definition C, ≥ 2 NE diagnosis within 3 years) is not adequate for this specific indication. It can thus be postulated that the most likely prevalence of NE in German claims data is 0.30% in 2019, which corresponds to approximately 250,000 affected individuals in Germany.

NE is a variant of eczema that presents with a co‐dominant type 3 immunity and is clinically distinct from typical AD. However, in practice overlapping diseases can be found in some patients.^9^, ^15^ In the current claims data, only a smaller proportion (about 18%) of individuals with NE were also coded for AD. A comparable pattern is observed in individuals diagnosed with AD. In this cohort, 1.76% of individuals were diagnosed with NE within a two‐year period preceding or following an AD diagnosis compared to 0.26% in the general population. The occurrence of other forms of eczema and dermatitis in NE was also below 10%. Since a certain degree of underrating cannot be ruled out given that some patients with NE may have been coded as AD, we also investigated the concurrence of NE with other atopic diseases. As expected, a correlation was found between NE and allergic rhinitis and allergic asthma, but this correlation was less pronounced than that between AD and NE.

Careful interpretation of these findings indicates that a distinction can be made between the AD and NE based on claims data with some overlaps, as evidenced in other sources.^3^, ^7^, ^10^ In total, these data like other publications underline the great need for more precise definition of NE both on clinical and molecular levels, consecutively, more precise clinical decision making may be needed, in particular among non‐dermatologists. However, further primary studies incorporating clinical data are required to enable a clearer distinction between NE and AD, as well as other eczematous diseases.

The results also confirm a high prevalence of NE in younger children which may be particularly confounded with AD. Moreover, in adults an increasing NE prevalence with age was observed as described in literature.^5^ Men are typically affected later in life, from around 50 years onward, whereas women are more often affected at a younger age, between 10 and 40 years, as shown in a previous US study.^7^ In addition to age‐related differences, regional variations were observed, with a particularly high prevalence in Saxony‐Anhalt and an unexpectedly low prevalence in Thuringia. Previous analyses have shown a higher overall morbidity burden in the eastern federal states.^16^ For this reason, the comparatively low prevalence of NE in Thuringia is particularly striking and requires further investigation.

### Strength and limitations

The fundamental strength of this analysis is the large number of persons in the SHI data and the high population coverage since about 90% of the German population is part of the statutory health insurance system.^17^ However, some limitations need to be considered when interpreting the results.

The populations of the various health insurance funds display considerable variations.^18^ In order to mitigate these discrepancies, the prevalence and incidence rates were standardized by age, sex and federal state to the German population. With the respect to external validity, a study on another dermatological disease (psoriasis) demonstrated that the epidemiological findings of the DAK‐G data can be extrapolated without limitation to the SHI population, provided that they are standardised.^11^ However, the findings may have limited generalizability to the entire population, as claims data do not include privately insured or uninsured individuals.

In general, claims data are collected for billing purpose and not for research objectives. Hence, the data only contain services that are reimbursed by the SHI system. Consequently, there is no information on health services which are paid by the individuals themselves or by third parties. SHI claims data may underestimate the true epidemiology, as conditions not leading to health care utilization are not captured. Furthermore, the data do not provide information on whether and when people actually take their prescribed medications after obtaining them from a pharmacy. This could be due to a number of factors, including insufficient or inadequate differential diagnosis, misclassification, or the coding behavior of the physician. The accuracy of coding eczematous diseases in routine care is uncertain, particularly due to variability in dermatological training and experience. In the case of NE, the uncertainties surrounding its etiology and pathophysiology, in conjunction with the absence of universally accepted diagnostic criteria, have the potential to further contribute to inconsistent coding. Furthermore, undercoding of NE in favor of AD may occur because a greater number of treatments are approved and reimbursed under the latter diagnosis, which could introduce systematic bias into claims‐based prevalence estimates.

In addition, claims data do not include clinical information on disease severity, quality of life, or detailed personal information such as weight, lifestyle, and education.^17^ It is important to recognize that the surrogate parameters used to quantify a severe form are only approximations of the true extent. However, the data suggests that the severity of the disease may be somewhat overestimated, given that the medications under consideration (between 92% and 98% received a medication) are also used for other dermatological conditions such as AD.

## CONCLUSIONS

This study emphasizes the challenges to accurately estimating the prevalence and incidence of NE due to the use of different case definitions based on performance data. Furthermore, this underscores the need for a harmonized guideline to improve both the diagnosis and treatment of individuals with NE, thereby leading to more accurate prevalence and incidence estimates.

## STANDARDS AND ETHICS

The study was conducted in accordance with the tenets of the Declaration of Helsinki. We considered the STROBE and STROSA statements and the criteria of a national good practice guideline.^17^, ^19^ According to the Good Practice of Secondary Data Analysis, a national guideline for the use of administrative databases, ethical committee approval is not required.^20^, ^21^

## FUNDING

The project was financially supported by Almirall. This was an independent, investigator‐initiated study supported by Almirall. Almirall had no role in the design, analysis or interpretation of the results in this study.

## CONFLICT OF INTEREST STATEMENT

Dr. Kristina Hagenström, Theresa Klinger, Katharina Müller and Charlotte Willers declare no conflicts of interest. Prof. Matthias Augustin has served as a consultant, lecturer, researcher, and/or has received institutional research grants from companies manufacturing drugs for eczema, including AbbVie, Almirall, Beiersdorf, Eli Lilly, Galderma, LEO, Novartis, Pfizer and Sanofi.

## Supporting information



Supplementary information

Supplementary information

Supplementary information

Supplementary information

Supplementary information
