# Supplementary material for: Epidemiology of nummular eczema – methodological approaches and outcomes from nationwide claims data analyses
Source: J Dtsch Dermatol Ges. 2025 Nov 16;24(7):886–93. doi: 10.1111/ddg.15932 (PMC13340949; doi:10.1111/ddg.15932)
Supplement: Supplementary file 1 — Supplementary information [file DDG-24-886-s004.docx]

Supplement Table S1 Prevalence of skin diseases and atopic conditions coded concomitantly among people with NE (base case definition A, ≥ 1 diagnosis of NE (N = 6,431)) compared to people without NE (N = 2,369,694) in 2022

|  |  | **NE** | | **Without NE** | |  |
| --- | --- | --- | --- | --- | --- | --- |
|  | **ICD-10-GM** | **n** | **%** | **n** | **%** | **Rate Ratio (95 % CI)** |
| Exfoliative dermatitis | L26 | 3 | 0.05 | 98 | 0.00 | 10.98 (3.60–33.48) |
| Lichen simplex chronicus | L28 | 195 | 3.03 | 7,431 | 0.31 | 9.71 (8.44–11.18) |
| Irritant contact dermatitis | L24 | 56 | 0.87 | 2,117 | 0.09 | 9.60 (7.40–12.44) |
| Unspecified contact dermatitis | L25 | 134 | 2.08 | 7,098 | 0.30 | 6.97 (5.88–8.26) |
| Seborrhoeic dermatitis | L21 | 378 | 5.88 | 24,141 | 1.02 | 5.99 (5.40–6.64) |
| Pruritus | L29 | 588 | 9.14 | 40,595 | 1.71 | 5.71 (5.24–6.21) |
| Atopic dermatitis | L20 | 1,155 | 17.96 | 93,997 | 3.97 | 5.25 (4.93–5.59) |
| Allergic contact dermatitis | L23 | 398 | 6.19 | 31,259 | 1.32 | 4.89 (4.42–5.40) |
| Dermatitis due to substances taken internally | L27 | 71 | 1.10 | 6,505 | 0.27 | 4.02 (3.19–5.08) |
| Alopecia areata | L63 | 45 | 0.85 | 5,280 | 0.22 | 3.22 (2.40 - 4.31) |
| Diaper dermatitis | L22 | 61 | 0.95 | 10,027 | 0.42 | 2.25 (1.75–2.89) |
| Allergic rhinoconjunctivitis | J30 | 891 | 13.85 | 195,810 | 8.26 | 1.78 (1.66–1.91) |
| Allergic Asthma | J45 | 762 | 11.85 | 196,355 | 8.29 | 1.49 (1.38–1.60) |
