# Supplementary material for: Epidemiology of nummular eczema – methodological approaches and outcomes from nationwide claims data analyses
Source: J Dtsch Dermatol Ges. 2025 Nov 16;24(7):886–93. doi: 10.1111/ddg.15932 (PMC13340949; doi:10.1111/ddg.15932)
Supplement: Supplementary file 2 — Supplementary information [file DDG-24-886-s002.docx]

Supplement Table S2 Systemic pharmaceutical drugs in NE and AD care observed in the current analysis

| **Drug group** | **Active ingredient** | **ATC** |
| --- | --- | --- |
| Systemic biologics | Dupilumab | D11AH05 |
|  | Tralokinumab | D11AH07 |
| Janus kinase inhibitors | Baricitinib | L04AA37 (until 2023) |
|  | Upadacitinib | L04AA44 (until 2023) |
| Systemic non-biologics  (conventional) | Methotrexate | L01BA01, L04AX03, M01CX01 |
|  | Mycophenolic acid | L04AA06 |
|  | Alitretinoin | D11AH04 |
|  | Azathioprine | L04AX01 |
|  | Ciclosporin | L04AD01 |
|  | Methoxsalen | D05BA02 |
|  | Trioxysalen | D05BA01 |
| Systemic Glucocorticosteroids (GCS) | Glucocorticosteroids | H02AB |
|  | - Betamethasone depot | H02AB51 |
|  | - Methylprednisolone depot | H02AB54 |
|  | - Prednisolone depot | H02AB56 |
|  | - Triamcinolone depot | H02AB58 |
