# Supplementary material for: Epidemiology of nummular eczema – methodological approaches and outcomes from nationwide claims data analyses
Source: J Dtsch Dermatol Ges. 2025 Nov 16;24(7):886–93. doi: 10.1111/ddg.15932 (PMC13340949; doi:10.1111/ddg.15932)
Supplement: Supplementary file 3 — Supplementary information [file DDG-24-886-s001.docx]

Supplement Table S3 Prevalence of severe nummular eczema (NE) diagnosis (dx) (≥ 1 sick leave, ≥ 1 hospital stay, ≥ 1 medication prescription) according the different case definitions from 2016 to 2022; “severe NE” was defined by either ≥ 1 sick leave, or ≥ 1 hospital stay, or ≥ 1 systemic drug prescription related to NE)

| **Year** | **Case definition** | **Insured persons with NE (N)** | **Severe NE, n (%)** | **Systemic drug, %** | **Sick leave, %** | **Inpatient stay, %** |
| --- | --- | --- | --- | --- | --- | --- |
| 2016 | A, ≥ 1 NE dx | 7,643 | 1073 (14.04) | 94.22 | 2.33 | 5.68 |
|  | B, ≥ 2 NE dx within one year | 1,959 | 313 (15.98) | 94.25 | 1.60 | 6.71 |
| 2017 | A, ≥ 1 NE dx | 7,487 | 1057 (14.12) | 95.18 | 2.74 | 5.11 |
|  | B, ≥ 2 NE dx within one year | 1,896 | 295 (15.56) | 95.59 | 2.71 | 4.75 |
| 2018 | A, ≥ 1 NE dx | 7,189 | 998 (13.88) | 95.49 | 1.70 | 5.31 |
|  | B, ≥ 2 NE dx within one year | 1,895 | 290 (15.30) | 94.48 | 2.41 | 6.55 |
|  | C, ≥ 2 NE dx within three years | 1,949 | 316 (16.21) | 92.09 | 2.85 | 9.81 |
| 2019 | A, ≥ 1 NE dx | 7,089 | 1038 (14.64) | 94.70 | 2.50 | 5.01 |
|  | B, ≥ 2 NE dx within one year | 1,865 | 327 (17.53) | 94.19 | 3.36 | 4.28 |
|  | C, ≥ 2 NE dx within three years | 2,822 | 478 (16.94) | 93.51 | 2.51 | 6.28 |
| 2020 | A, ≥ 1 NE dx | 6,544 | 862 (13.17) | 96.40 | 1.86 | 3.95 |
|  | B, ≥ 2 NE dx within one year | 1,727 | 254 (14.71) | 95.28 | 2.37 | 4.35 |
|  | C, ≥ 2 NE dx within three years | 2,858 | 435 (15.22) | 94.48 | 2.31 | 6.24 |
| 2021 | A, ≥ 1 NE dx | 6,829 | 938 (13.74) | 96.27 | 1.28 | 4.16 |
|  | B, ≥ 2 NE dx within one year | 1,805 | 298 (16.51) | 97.32 | 0.67 | 3.69 |
|  | C, ≥ 2 NE dx within three years | 2,938 | 451 (15.35) | 95.79 | 0.89 | 5.54 |
| 2022 | A, ≥ 1 NE dx | 6,431 | 949 (14.76) | 97.37 | 1.48 | 3.48 |
|  | B, ≥ 2 NE dx within one year | 1,689 | 283 (16.76) | 98.23 | 1.41 | 3.89 |
|  | C, ≥ 2 NE dx within three years | 2,779 | 424 (15.26) | 97.64 | 1.65 | 4.01 |
